# Semi-Hybrid CO_2_ Laser Metal Deposition Method with Inter Substrate Buffer Zone

**DOI:** 10.3390/ma14040720

**Published:** 2021-02-04

**Authors:** Bogdan Antoszewski, Hubert Danielewski, Jan Dutkiewicz, Łukasz Rogal, Marek St. Węglowski, Krzysztof Kwieciński, Piotr Śliwiński

**Affiliations:** 1 Laser Research Centre, Faculty of Mechatronics and Mechanical Engineering, Kielce University of Technology, Al. Tysiąclecia P.P. 7, 25-314 Kielce, Poland; hdanielewski@tu.kielce.pl; 2 Institute of Metallurgy and Materials Science, Polish Academy of Sciences, PAS, 25, Reymonta St., 30-059 Krakow, Poland; dutkiewicz.j@imim.pl (J.D.); rogal.l@imim.pl (Ł.R.); 3 Lukasiewicz Research Network-Instytut Spawalnictwa (Institute of Welding), 16-18 Bl. Czesława Str., 44-100 Gliwice, Poland; marek.weglowski@is.gliwice.pl (M.S.W.); krzysztof.kwiecinski@is.gliwice.pl (K.K.); piotr.sliwinski@is.gliwice.pl (P.Ś.)

**Keywords:** direct laser deposition, metal powder, filler wire, semi-hybrid method, additive manufacturing, microstructure analysis

## Abstract

This article presents the results of the metal deposition process using additive materials in the form of filler wire and metal powder. An important problem in wire deposition using a CO_2_ laser was overcome by using a combination of the abovementioned methods. The deposition of a multicomponent alloy—Inconel 625—on a basic substrate such as structural steel is presented. The authors propose a new approach for stopping carbon and iron diffusion from the substrate, by using the Semi-Hybrid Deposition Method (S-HDM) developed by team members. The proposed semi-hybrid method was compared with alternative wire and powder deposition using laser beam. Differences of S-HDM and classic wire deposition and powder deposition methods are presented using metallographic analysis, within optic and electron microscopy. Significant differences in the obtained results reveal advantages of the developed method compared to traditional deposition methods. A comparison of the aforementioned methods performed using nickel based super alloy Inconel 625 deposited on low carbon steel substrate is presented. An alternative prototyping approach for an advanced high alloy materials deposition using CO_2_ laser, without the requirement of using the same substrate was presented in this article. This study confirmed the established assumption of reducing selected components diffusion from a substrate via buffer layer. Results of metallographic analysis confirm the advantages and application potential of using the new semi-hybrid method for prototyping high alloy materials on low alloy structural steel substrate.

## 1. Introduction

Recent years have seen the rapid development of Additive Manufacturing (AM) technologies. The high application potential in advanced industries has encouraged many researchers to study these processes. The additive process, particularly laser metal deposition combining unique properties of laser beam and potential of building metal components, allows developing advanced structures with good properties [1]. The deposition process can be performed using different methods, where additional material is conveyed to the area of laser beam interaction with a substrate: Laser Engineering Net Shaping (LENS), Direct Laser Deposition (DLD), Laser Metal Deposition (LMD), etc., or additional material laying on substrate is molten by laser beam: Selective Laser Sintering (SLS), Laser Powder-Bed Fusion (LPBF), etc. [2,3]. In the prototyping process of high alloy materials such as Inconel 625—a nickel based super alloy with high temperature resistance and corrosion resistance, dedicated to marine, nuclear, energetic, and aerospace applications—it is important to achieve good properties, uniform structure, and a lack of defects, which may influence material characteristic [4]. The fusion mechanism during prototyping affects those aspects; moreover, thermal cycles and diffusion of alloying elements from substrate to deposited layers or vice versa have a great impact on the manufacture material [5]. Generally, in the first group of prototyping methods, substrate has a chemical composition similar to that of the deposited material, in order to obtain uniform composition and properties. Considering prototyping of advanced high alloy materials, cost of the substrate even in small series production, due to its thickness requirement, is substantial. Therefore, developing a method where less expensive material can be used is an important aspect. The requirement for identical materials can be omitted using the buffer zone, where generally the chemical composition of the first deposited layer differs from the substrate and the deposited material [6]. The buffer layer between the substrate and the prototyped material is generally deposited using the same method as further deposition; however, these techniques require the manufacturing of higher elements and cutting off some portion from the deposited material [7]. Moreover, additional alloying elements from the buffer zone can diffuse to the upper layers and may affect their properties. Depending on the deposited material’s form (powder or filler wire), the density of the manufactured material will be slightly different. Lower power density and temperature absorbed by the substrate occur during deposition of metal powders; however, density of the developed material is lower than that in the wire deposition method. By using fiber laser, precise melting of additional materials can be obtained. It is more problematic when we consider prototyping with a filler wire and CO_2_ laser, where bonding with the substrate is obtained by using deep material penetration via the keyhole effect [8]. High mixing of alloying elements between the substrate and the deposited material appears when a keyhole penetrates the substrate. Nevertheless, reflectivity of the metallic surface for CO_2_ lasers is too high for a bonding substrate with deposited layers without this effect. Therefore, this type of AM process is characterized as problematic and requires a specific approach. A preliminary study shows that prototyping with a filler wire gives better material density, which is relevant in some particular applications; therefore, the authors have focused on the development of this prototyping method [9]. Some applications use a combination of two different deposition methods; however, these hybrid methods are used simultaneously and affect only process efficiency and fail to solve the problem of diffusion between the substrate and deposited layers [10,11]. An alternative solution of using the buffer interlayer zone is applied in the S-HDM process proposed by the authors—i.e., a combination of two deposition methods, where the first two layers are deposited using additional material in the form of metal powder, and after the material is cooled (temperature decrease to normal conditions), proper layers are deposited using a laser beam and filler wire. High alloying components’ diffusion, accompanying the pure wire deposition method is restrained by the buffer zone made of metal powder and does not reach the substrate; therefore, good properties are provided from the first layer. The properties of the deposited material are related to the thermal cycles and chemical composition of the used materials. In the first case, the deposition of additional material in the form of filler wire was performed directly on the substrate; in the second case, metal powder was deposited directly on the substrate, while in the third case, filler wire was deposited on a buffer layer, developed by melting metal powder on a substrate surface. Mixing of the alloy components of the substrate and additional materials for the analyzed methods is different; therefore, metallographic analysis is required. Micro and macrostructure study was carried out using Scanning Electron Microscopy (SEM) and diffusion of selected alloying elements was tested with Energy Dispersive Spectroscopy (EDS) analysis. A visual microscopic test was carried out to confirm the quality of the manufactured materials and detect potential defects in the deposited material. The metallographic structure of the substrate, buffer, and deposited zone were tested. The prototyping of high alloy material on a substrate with different chemical composition is complex. The diffusion of alloying elements affects the structure and changes the properties of the developed material [12,13,14]. Moreover, the mixing process can produce precipitations. Therefore, to confirm a uniform structure, especially in the semi-hybrid method, qualitative and quantitative analysis was carried out, using energy-dispersive X-ray spectroscopy with a scanning electron microscope JSM-7100F (JEOL Ltd., Tokyo, Japan).

## 2. Methodology

### 2.1. Materials

Low-carbon structural steel, grade S235JR, in the form of a 6-mm sheet was used as a substrate material. The commonly used low-carbon steel served as a substrate to show the effect of stopping carbon and iron migration to the upper deposited layers [15]. The S235JR is an unalloyed steel with carbon content up to 0.2% and a trace amount of other alloying elements (Table 1). The material used for deposition was a nickel-based super alloy Inconel 625 in the form of a powder and filler wire, and with similar composition (Table 1). Inconel 625 has great heat and corrosion resistance; however, when carbon precipitations appear, intergranular corrosion can occur and lead to a decrease in those properties [16].

The structure of S235JR steel is typically ferritic-pearlitic. Low carbon content and trace amounts of alloying elements reduce steel hardness; however, some strengthening effect may occur through steel phase transformations. Inconel 625 has a typical austenitic structure with high content of nickel (over 60%), chromium (more than 20%), and elevated amounts of molybdenum and niobium. High content of these elements affect heat and corrosion resistance properties as well as good mechanical characteristics [17,18]. For the deposition process, Inconel 625 was used in the form of spheroidal gas atomized powder, with nominal particle size 90 + 45 µm (according to inspection certification provided by manufacturer Oerlikon Metco, Pfäffikon, Switzerland), and filler wire OK Autrod NiCrMo-3 (Esab, Gothenburg, Sweden) with a diameter of 1 mm.

### 2.2. Experimental Procedure

**Figure 1:**
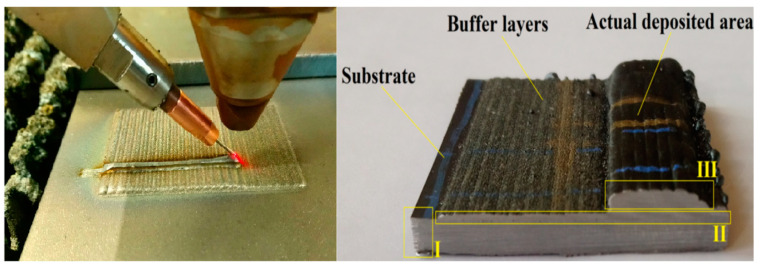
Deposition and the developed specimen using the semi-hybrid method.

The experimental procedure was divided into three separate stages. For every part, Trumpf CO_2_ laser integrated with TruLaserCell 1005 machine (Trumpf, Ditzingen, Germany) was used to perform the deposition process. The first experiment concerned deposition of Inconel 625 alloy in the form of filler wire on the S235JR substrate. The second experiment shows powder deposition of Inconel 625 on the S235JR substrate. The third experimental procedure was divided into two steps: first, the substrate material was cladded using melting of metal powder, depositing a two-layer buffer zone. The developed zone consists of clads overlapping each other at half its width. In the second step, few layers using filler wire and CO_2_ laser beam were deposited on the developed buffer layer (Figure 1). In order to reduce the plasma ionization effect, helium (5.0) with a flow rate equal to 20 L/min was used as a shielding gas, and for deposition of metal powder, argon was used as a conveying gas.

Prototyping processes were performed using two process heads: a welding head with focal length equal to 270 mm and a side wire feeder as well as a cladding head with focal length equal to 250 mm and coaxial metal powder delivery system GTV M-PF 2/2. Deposition of metal powder on the substrate surface (Figure 1, zone I) for powder deposition and S-HDM processes were performed with output power equal to 2.25 kW, process head speed ratio 0.8 m/min, and interval of single layer 2.5 mm. The powder feed rate equal to 15 g/min resulted from a rotation disc dispenser speed equal to 5 rpm and conveyed gas flow 5 l/min. For the filler wire deposition, output power was set to 3 kW, with process speed equal to 1.5 m/min, and similar wire feed rate equal to 1.5 m/min, oriented at an angle of 45 degrees [19]. However, the deposition of buffer layers (Figure 1, zone II) changes the initial conditions for wire deposition (Figure 1, zone III); therefore, a lower output power equal to 2.8 kW with deposition speed (both process head and wire feed rate) was increased to 2 m/min for the first two layers. For the upper layers, starting from the third layer, the parameters were equal to the wire deposition process alone. Changing of parameters was performed in order to unify the lower layers by the re-melting process.

## 3. Results

### 3.1. Global Observation of the Obtained Inconel 625 Specimens

**Figure 2:**
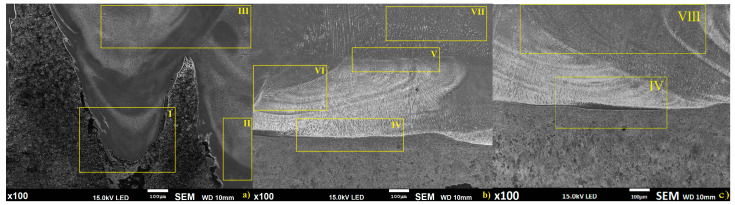
Macroscopic results of the deposited materials using with defined characteristic areas for: (**a**) laser wire deposition method, (**b**) semi-hybrid method, (**c**) laser powder deposition method.

The obtained specimens have similar dimensions; however, the structures of clad bead geometries for the three mentioned methods are significantly different (Figure 2).

Global observation showed some characteristic regions for analyzed deposition methods, where, for the wire alone method (Figure 2a), three separate regions were identified: I—penetration zone of wire deposition, II—zone of non-uniform structure and mixing of alloy components, III—wire deposition layers overlap zone. For the semi-hybrid method (Figure 2b), four separate regions were identified: IV—fusion line of powder layer and substrate, V—fusion zone between the buffer layers and wire deposited layers, VI—zone of wire penetrated buffer layers, VII—pure wire deposited zone. For the powder deposition method, two separate regions were identified: IV—fusion line of powder layer and substrate (similar for the region IV in S-HDM), VIII—pure powder deposited zone [20]. Moreover, the fusion mechanisms in the deposition process are different for all considered methods, i.e., for the first method, deep penetration in the substrate occurs, while for the second method, deep penetration occurs only inside the buffer layer and does not reach the substrate material, and for the third method, fusion mechanism appears alongside the substrate surface [21]. The microstructure of the obtained specimens showed significant differences; therefore, further metallographic study of the characteristic region defined in Figure 2 is required and is presented in the next paragraph.

### 3.2. Metallographic Analysis

**Figure 3:**
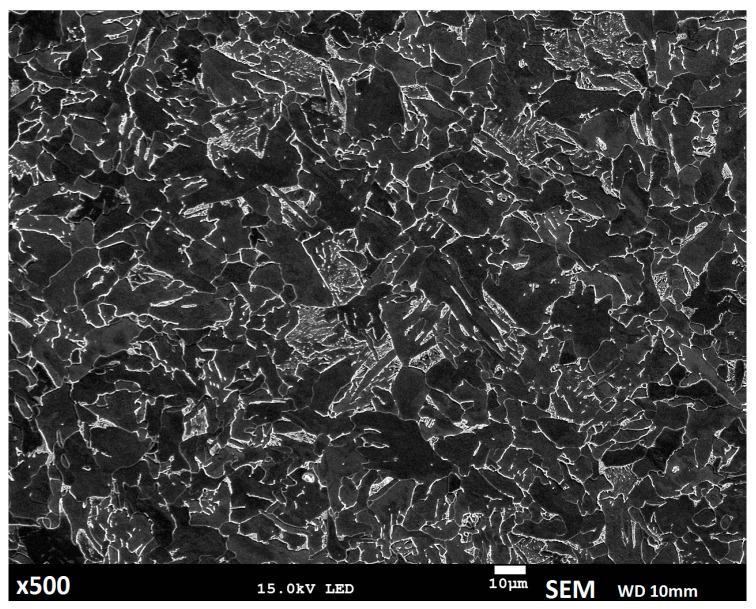
Microstructure of the substrate material.

Optical and electron microscopes were used for metallographic structure analysis. The substrate material has a fine-grain, ferritic-pearlitic (dark areas ferrite and bright pearlite) structure, typical for low-carbon steel, without explicit visible banding (Figure 3).

**Figure 4:**
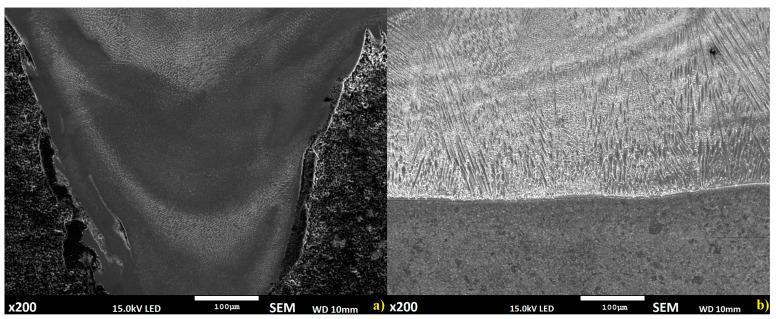
Structure of fusion zone between the substrate and deposited layers: (**a**) laser wire deposition method, (**b**) semi-hybrid and powder deposition methods.

The fusion mechanisms of the deposition methods being investigated are different; in wire or powder deposition is only one type of thermal process related to keyhole effect or over melting substrate with molten powder (Figure 4a). However, for the proposed semi-hybrid deposition method, two different thermal processes occur, first when metal powder is deposited on the substrate surface and second with keyhole effect. Fusion of the first layers in the S-HDM process is highly limited to the fusion line between the molten powder and the substrate (Figure 4b) [22]. According to global observation, certain separate regions for all the mentioned methods can be identified. Microstructure in the material manufactured using the wire deposition method is complex, changing according to specimen height; differences in dendritic growth can be observed in all methods [23]. However, in the proposed S-HDM, a more uniform structure occurs, with differences visible only in dendritic growth direction; a similar phenomenon was observed in the powder deposition method. For the analyzed methods, the upper layers reveal differences only in dendritic growth direction related to each padding bead and dendrite size [24].

**Figure 5:**
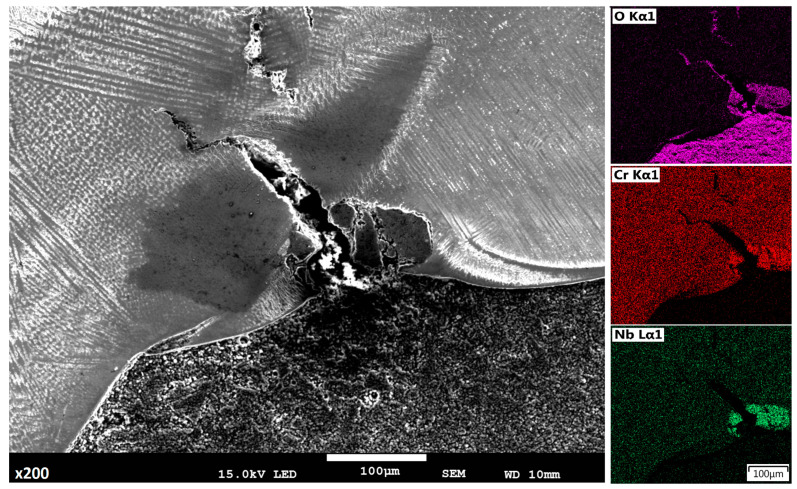
Defect alongside the fusion line, between the substrate and deposited layers in the wire deposition method.

Differences in the mechanism of deposition process affect the mixing factor between the substrate and deposited layers. In the first case, a non-uniform complex structure appears, while in the second and third case, no relevant differences in structure were identified. High temperature gradient in the wire deposition method, considering situation when the substrate material has a different chemical composition and properties than that of the deposited material, can lead to defects related to solidification and inclusion phenomena (Figure 5) [25,26,27]. The inclusion was determined by using energy-dispersive X-ray spectroscopy performed with a JSM-7100F scanning electron microscope. The results suggest formation of niobium oxide at the interface.

**Figure 6:**
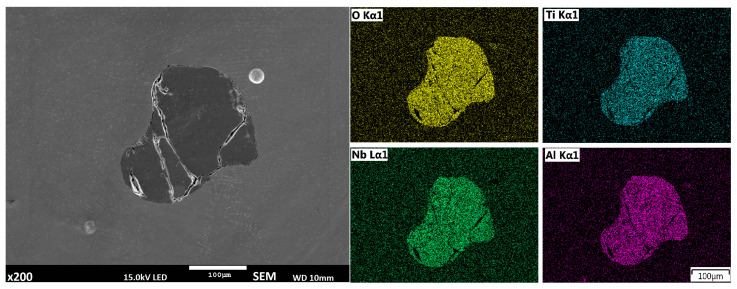
Inclusion inside the buffer zone, deposited using laser melting of metal powder.

Defects occur in the material not only in the wire or powder deposition methods; some inclusions are identified in the developed semi-hybrid method as well. However, in this case, they are revealed in the buffer layer, and are related to the oxidation process (Figure 6); they are most probably (Ti,Nb,Al)_x_O_y_ type oxides [28].

**Figure 7:**
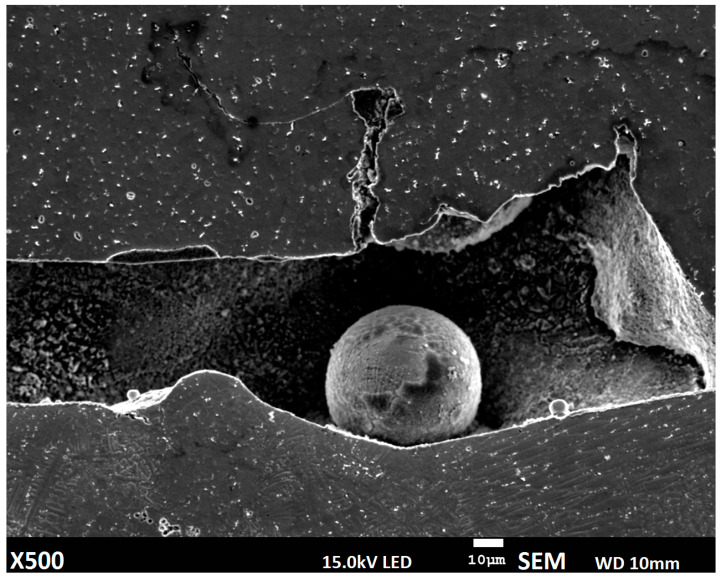
Discontinuities in material manufactured using the powder deposition method.

Deposition of metal powder alone provided good properties with restriction of chemical components from the substrate; however, the deposition mechanism can lead to discontinuities in the material due to disturbances in the solidification process (Figure 7).

Deposition of metal powder, where additional material is conveyed using inert gas, combined with limited absorption of CO_2_ laser radiation for metals, lead to forming a cavity with partially molten powder particles. This phenomenon, along with porosity caused by gas flow, is well known and the main reason of material density limitation for the powder deposition process.

**Figure 8:**
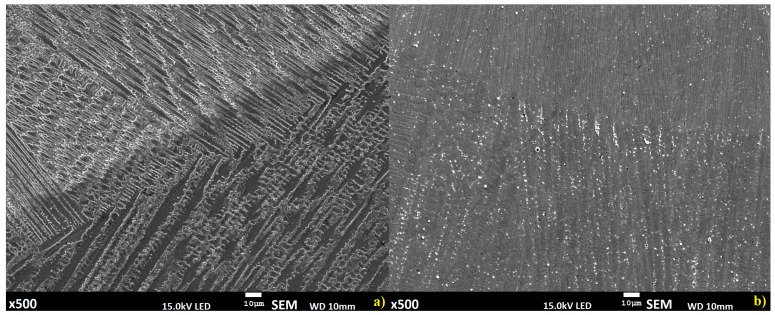
Microstructure of the interlayer zone between the second and third layers for: (**a**) wire deposition method, (**b**) semi-hybrid deposition method.

The microstructure of the deposited layers is complex, a predominantly dendritic structure is identified; however, dendrites growth is related to individual layer deposition (Figure 8) [29].

**Figure 9:**
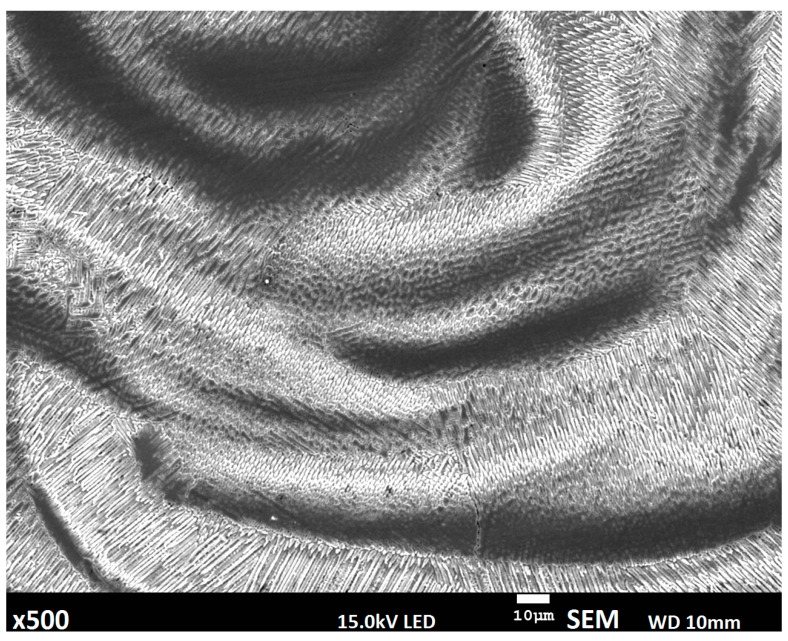
Structure of fusion zone obtained during deposition of filler wire directly on the substrate.

The presented interlayer structures are similar; however, the direction of dendritic growth reveals some differences related to the differences of fusion between wire to wire, powder to powder, and wire to powder deposition. Microstructure of the upper layers is similar and consists of pillar-dendritic structure [30,31]. However, in the bottom layers alongside the fusion line, some differences are identified, and they mostly appear in the material developed using the wire deposition alone method (Figure 9).

The manufactured materials have a dominating pillar-dendritic structure; however, in fusion regions in this method, regions with a cellular dendritic structure can also be observed [32].

**Figure 10:**
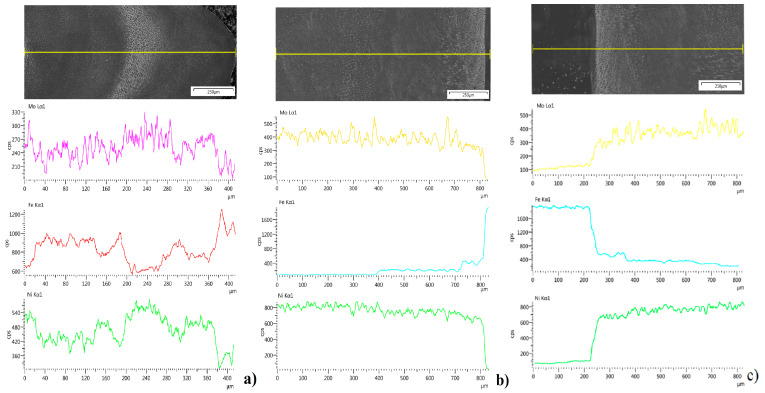
Distribution of Mo, Fe, and Ni in the fusion area: (**a**) wire deposition, (**b**) semi-hybrid deposition, (**c**) powder deposition method.

A chemical composition analysis of the fusion region where deposited materials are mixed with the substrate was performed (Figure 10) based on the chemical composition of those materials (Table 1). Using iron, nickel, and molybdenum identification in the fusion zone, diffusion of the alloying elements from the substrate to deposited layers was analyzed [33].

Distribution of the measured alloying elements shows a higher mixing factor for S-HDM; meanwhile, in wire deposition, non-uniform distribution is reveled. The deposition method using filler wire without a buffer zone affected the appearing of local areas, where both the substrate and deposited material are not fully mixed, and clear boundaries can be indicated [34]. The buffer zone used in S-HDM makes it possible to stop the migration of alloying elements from the substrate; this is clearly shown for iron distribution.

Differences in iron concentration between the second and third layers reveal a more intense migration of iron from the substrate to the upper layers for the deposition method using filler wire alone, while for the developed semi-hybrid method, iron diffusion is limited to the buffer zone, similar to the powder deposition method.

## 4. Discussion

The materials used for deposition have important differences in chemical composition compared to the substrate material (Table 1). The aforementioned differences affect diffusion and solidification processes, which is problematic, especially when we consider deposition nickel based alloy on substrate made of low-carbon steel.

Deposition parameters were developed according to a separate preliminary study. Therefore, for the filler wire deposition method, output power was equal to 3 kW with deposition speed (movement of process head, similar to wire feed rate) of 1.5 m/min for wire deposition alone. However, those parameters were corrected, and for deposition of the first two layers for the semi-hybrid method, output power reduction was performed (to 2.8 kW), and deposition speed was increased (to 2 m/min). The buffer layer as well as powder deposition method was obtained with output power equal to 2.25 kW and deposition speed combining process head movement speed of 0.8 m/min and powder delivery feed rate of 15 g/min.

Global observation showed significant differences in the fusion zone between the substrate and the deposited materials depending on the deposition process. Moreover, macroscopic analysis showed a potentially non-uniform structure in the fusion zones for the wire deposition method and cavity with partially molten powder for the powder deposition method [35]. Therefore, in order to investigate the quality level of the deposition process, and compare filler wire and powder alone with the semi-hybrid deposition method developed by the authors, metallographic studies were carried out. According to identified separate regions (Figure 2) microstructure and chemical elements distribution study was conducted. Region I presented in Figure 2 shows a fusion zone deep inside the substrate material obtained via keyhole penetration. The alloy component mixing from the substrate and deposited material are high; moreover, the obtained microstructure is not uniform, and clear boundaries for wire deposition alone can be identified (Figure 9).

Prototyping using the proposed new semi-hybrid method combines advantages of powder and wire deposition, where the fusion zone of the substrate and deposited material is narrow, and is related to the thin film of the substrate surface melted by deposited material in liquid state. This phenomenon allows obtaining a permanent bonding between the substrate and first layers of the deposited material; however, diffusion between them is limited [36]. In the proposed semi-hybrid method, deep penetration in wire deposition reaching the substrate is stopped by the buffer zone (Figure 2: regions V and VI). Microstructure of the manufactured materials is dendritic; however, dendritic orientation differs depending on the deposition method (Figure 8). For the upper layers obtained, structures are similar for all three analyzed methods, where a columnar structure can be observed. The microstructure of prototyped material is complex and is related to fusion and solidification processes. Microstructures of the deposited layers are composed of two regions: the bottom part of each layer is mostly a columnar structure where no secondary dendrites occur, and the top part of the layers shows typical fine dendritic structure with secondary dendrites. The cooling rate of the molten pool, and consequently the solidification velocity is higher at the bottom part of the layer and relatively slower at the top part. The bottom part of each layer consists of mainly primary dendrites, and because of the progressive decrease of the cooling rate from the bottom to the top layers, a gradual transition of microstructure from fully columnar to dendritic transition is observed, with the primary and secondary dendrites. Dendritic formation is related to the deposition mechanism, where in wire deposition via keyhole effect high thermal gradient occurs and leads to a faster nucleation process; therefore, a greater amount of dendrites is observed. Structure of the material manufactured by S-HDM is similar in the upper layers; however, in the first layer, due to a slower solidification process, longer dendrites can be observed. A similar situation can be observed in the powder deposition method [37].

The ferritic-pearlitic structure of the substrate material consisted of a substantial iron amount, and diffusion of this element combined with migration of carbon to Inconel 625 layers can reduce its properties, especially high temperature resistance and corrosion resistance. The wire deposition alone method via keyhole deep penetration affects intensive mixing of deposited material with the substrate, when the keyhole penetrates the bottom workpiece. The influence of the flow field is clearly evident from the corresponding solidified structure. In the case of substrate penetration, discrete growth bands occur in the entire solidified padding beads, suggesting severe fluctuations in the flow field and the growth process (Figure 9) [38].

All analyzed deposition methods are related to direct laser deposition of filler wire or metal powder; however, in the proposed semi-hybrid method, a combination of those two methods are presented, where additional powder deposition of the first two layers is performed in order to provide a buffer zone to restrict iron and carbon diffusion from the substrate. Deposition of high alloy material is associated with a complex structure formation and numerous inclusions related to the solidification process. In the classical wire deposition method, a greater thermal gradient appears alongside the substrate material, and differences between density and thermal properties of the substrate and deposited materials can lead to cracking in places where the keyhole penetrates the substrate (Figure 5) [39]. SEM analysis revealed that this defect is also related to inclusion of chromium and niobium oxide. In the developed S-HDM, some inclusion with more complex composition appears, where Titanium, Niobium, and Aluminum oxides are detected (Figure 6). The place of Ti,Nb,Al_x_O_y_ oxides detection was located in a buffer zone. This type of precipitation most likely appears in materials when temperature of the buffer zone reaches 700 °C (973 K). The γ” phase precipitates (A3 B type) and a tetragonal crystal structure begins to form, often in a disk-shaped morphology, coherent with respect to the matrix [40].

**Figure 11:**
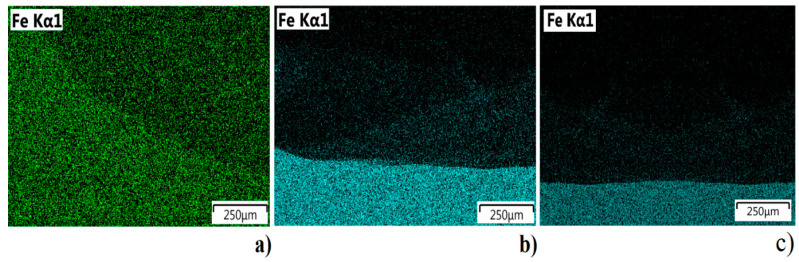
Iron distribution between the second and third layer for: (**a**) wire deposition, (**b**) developed S-HDM, (**c**) powder deposition.

The homogenous structure of manufactured materials is related to the uniform distribution of chemical elements. Therefore, an analysis of the defined regions in the cross-section was performed based on the linear and map distribution of Mo, Fe, and Ni. The performed study shows greater differences in the wire deposition method without a buffer zone. Distribution of alloying elements in the material deposited using the wire alone method fluctuates, and uniform composition and structure do not occur (Figure 10a). The material manufactured using the powder deposition method is homogenous (Figure 10c); however, some deposition defects in the form of gas pore and cavity can be observed (Figure 7). The specimen obtained using the semi-hybrid method shows two separate areas: area with low iron concentration (deposited buffer zone), where iron from the substrate material is bounded, and the area where only iron from the deposited material (Inconel 625 wire) occurs (Figure 10b). Iron distribution between the substrate, buffer zone, and deposited layers confirms the assumed restriction of elements from the substrate. Moreover, the distribution of iron between the first layers shows a higher migration in the wire deposition alone process [41]. Restriction of iron distribution using a buffer zone results in the uniform composition of the deposited upper layers, which means the improvement of properties as well (Figure 11). The analysis revealed considerable differences in diffusion mechanism related to bonding of the deposited material with the substrate. The proposed method allows restraining alloying elements diffusion from the substrate and makes it possible to obtain good, uniform chemical composition of the manufactured material from the very first layers deposited using the wire deposition method.

The obtained uniform composition and structure of the deposited material provide good mechanical properties, as well as corrosion and temperature resistance, which is crucial in power plant construction, especially for components of nuclear installation. While using the proposed method, reduction in material costs (of substrate and filler wire to manufacture additional layers intended to be cut off) can be achieved. While restraining of iron and carbon diffusion from the substrate can be obtained using powder deposition alone or proposed semi-hybrid methods, better material density can be provided by the wire deposition method. Even trace amounts of iron and carbon from the substrate or buffer zone affect the properties of the deposited material, which change with the subsequent layers. Applying a buffer zone with similar chemical composition allows to obtain materials with similar composition and properties starting from the first wire deposition layers. Moreover, the deposited additional buffer zone is bonded with the substrate using less power than in the wire deposition method; therefore, stress-strain concentration in the substrate and its deformation are reduced.

## 5. Conclusions

A new developed semi-hybrid deposition method, consisting of combining selective metal deposition of metal powder with deposition using filler wire was performed. The proposed method was compared with filler wire and metal powder deposition methods. The presented results show significant differences in bonding mechanism between the substrate and deposited materials. Deep material penetration via the keyhole effect leads to a high mixing ratio of deposited layers with the substrate. However, using S-HDM, where the first two layers are deposited using metal powder causes diffusion of alloy components from the substrate to be restrained, similar to the powder alone deposition method. Some inclusions occur for the three mentioned methods, i.e., wire deposition is related to solidification between the substrate and the lower layers, while for the proposed method, complex oxides containing Nb, Ti, and Al were identified in the deposited buffer zone. Combination of the two deposition methods makes it possible to overcome CO_2_ laser wire deposition disadvantages. The requirement of obtaining a keyhole effect with deep penetration affecting high mixing of the substrate and the deposited material was reduced by the buffer layer. Similar composition of the buffer and deposited layers allows the development of materials with good characteristics. This new approach to wire deposition and CO_2_ lasers enables the use of this type of machine and the wire deposition method to manufacture high alloy materials on the substrate made of inexpensive structural steel. The present study confirms the established assumption of reducing iron diffusion from inexpensive substrate by using buffer layers. The deposition of a two-layer buffer zone using selective melting of metal powder having a composition similar to that of the inexpensive filler wire makes it possible to use wire deposition and CO_2_ laser in order to manufacture an advanced structure of uniform composition and good properties. Further study of the developed S-HDM is planned and will be aimed at comparing the mechanical properties of materials prototyped using the proposed new method, filler wire deposition, and metal powder deposition methods.

## Figures and Tables

**Table 1:** Chemical composition of the used materials.

Material/Element (wt.%)	Mn	Si	Cu	Cr	Ni	Nb	Mo	B
S235JR	1.65	0.5	0.4	<0.15	<0.15	0.06	0.08	0.0008
Inconel 625	0.4	-	-	21.8	62.7	3.66	9.1	-

## Data Availability

Data sharing not applicable.
